# Differential association of moderate alcohol use with neurodegeneration, cerebrovascular disease, and cognition by racial, ethnic and sex/gender groups in middle age

**DOI:** 10.1002/alz.091479

**Published:** 2025-01-09

**Authors:** Diana S. Guzmán, Miguel Arce Rentería, Justina F Avila, Indira C. Turney, Aubrey S. Johnson, Anna C. Smith, Amarachukwu Okafor, Hannah M. Houlihan, Lauren B. Heuer, Mohamad J. Alshikho, Clarissa Morales, Jessica Mazen, Yian Gu, Jennifer J. Manly, Adam M. Brickman, Patrick J. Lao

**Affiliations:** ^1^ Columbia University Irving Medical Center, New York, NY USA; ^2^ Department of Neurology, Columbia University, New York, NY USA

## Abstract

**Background:**

Moderate alcohol use may be associated with brain and cognitive benefits compared to heavy alcohol use. However, results have varied. We hypothesized that the relationship between alcohol use and cognition is mediated by neurodegenerative and cerebrovascular measures in a diverse middle‐aged sample of non‐alcohol dependent community‐dwelling adults in Northern Manhattan.

**Method:**

Participants (N=451; age=53±10 years; 36 Black Men (BM), 68 Black Women (BW), 95 Latinx Men (LM), 198 Latinx Women (LW), 17 White Men (WM), 37 White Women (WW)) from the Offspring study of Racial and Ethnic Disparities in Alzheimer’s Disease underwent structural and vascular MRI (total brain volume (TBV), cortical thickness in AD signature regions, hippocampal volume, white matter hyperintensities (WMH)). Moderate alcohol use was assessed by the Alcohol Use Disorder Identification Test–Concise (AUDIT‐C≤9). Mediation models tested whether the association of alcohol use on cognition (SRT total and delayed; Animal and letter fluency; Digit Span Backwards) is mediated by brain measures, stratified in sex/gender by racial and ethnic groups.

**Result:**

Greater moderate alcohol use was associated with lower cortical thickness and hippocampal volume LM (cortical thickness: ‐0.005 mm [‐0.008, 0.001], p=0.005; hippocampal volume: ‐25.0 mm^3^ [‐45.0, ‐4.7], p=0.02), greater vascular burden in BM (temporal WMH: 0.07 cm^3^ [0.001, 0.121], p=0.05), and lower TBV in WM (‐24.0 cm^3^ [‐27.5, ‐20.4], p=0.01), but not associated with brain measures in women. Greater moderate alcohol use was associated with lower animal fluency for LM (ADE: ‐0.25 to ‐0.21, p≤0.04), but higher letter fluency for LW (ADE: 0.24 to 0.25, p≤0.04) and BW (ADE: 0.43 to 0.43, p≤0.05), accounting for brain to cognition associations. However, mediation pathways (ACME) were not significant.

**Conclusion:**

Men had lower cortical thickness and brain volume, greater WMH volume, and lower cognitive measures, while women had higher cognitive measures with greater moderate alcohol use in midlife. Neurodegenerative and vascular associations with moderate alcohol use differed by race and ethnicity in men, while the directionality of associations between moderate alcohol use and fluency differed by sex/gender. Future work should explore alcohol use factors (e.g., context of use, duration of use), and the relationship to brain and cognitive changes over the life course.

